# Video-assisted thoracoscopic lobectomy for a patient with rare partial anomalous pulmonary vein connection: Case report

**DOI:** 10.1097/MD.0000000000042461

**Published:** 2025-05-09

**Authors:** Xiayimaierdan Yibulayin, Guliniaer Feida, Xiaohong Sun

**Affiliations:** a Department of Thoracic Surgery, Affiliated Tumor Hospital of Xinjiang Medical University, Urumqi, China; b Department of Radiology, Affiliated Tumor Hospital of Xinjiang Medical University, Urumqi, China.

**Keywords:** cancer, lobectomy, partial anomalous pulmonary vein connection, pulmonary sarcomatoid carcinoma

## Abstract

**Rationale:**

Partial anomalous pulmonary venous connection (PAPVC) is a rare congenital vascular anomaly that often remains undetected in asymptomatic patients. While most cases are hemodynamically insignificant, failure to recognize PAPVC preoperatively may lead to intraoperative misidentification and surgical complications. Coexistence of PAPVC and lung cancer, particularly on the ipsilateral side, is extremely rare and poses challenges for surgical decision-making.

**Patient concerns:**

A 55-year-old man, asymptomatic and uncharacteristic in his past medical history, presented with an abnormal shadow in the left lower lobe during a regular checkup.

**Diagnoses:**

Pulmonary sarcomatoid carcinoma, partial anomalous pulmonary vein connection.

**Interventions:**

The patient underwent video-assisted thoracoscopic left lower lobectomy without surgical correction of the PAPVC.

**Outcomes:**

The patient remained in good health at the 14-month follow-up.

**Lessons:**

In patients with ipsilateral PAPVC and no evidence of hemodynamic compromise, anatomical lobectomy alone may be a safe and effective surgical option without the need for venous correction.

## 1. Introduction

Partial anomalous pulmonary venous connection (PAPVC) is an uncommon congenital anomaly, occurring in approximately 0.4% to 0.7% of the population.^[1]^ In most asymptomatic individuals, it has no significant hemodynamic consequences. Nearly half of PAPVC remain undiagnosed before surgery and are often not detectable on plain radiographic studies.^[2]^ Lung cancer, a leading cause of cancer-related death globally, has rarely been reported in association with PAPVC. When PAPVC is located in the contralateral lung lobe, performing an extensive pneumonectomy without prior correction of the anomaly may result in poor outcomes due to significant left-to-right shunting.^[3]^ However, PAPVC is seldom encountered during routine lobectomy, and its preoperative recognition remains a clinical challenge. In this report, we present a rare case of pulmonary sarcomatoid carcinoma coexisting with ipsilateral PAPVC, which was successfully managed with video-assisted thoracoscopic left lower lobectomy.

## 2. Case presentation

**Figure 1.**
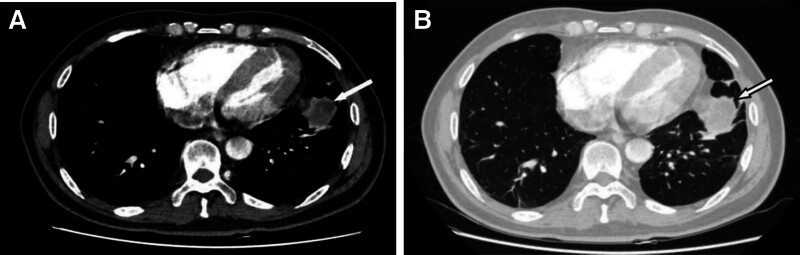
CT showed a mass in the left lower lobe. (A) The white arrow in the mediastinum window points to a mass in the left lower lobe, which has enlarged with marked heterogeneous enhancement and central necrosis. (B) The lung window reveals poorly defined margins and a concave adjacent pleural retraction. CT = computed tomography.

**Figure 2.**
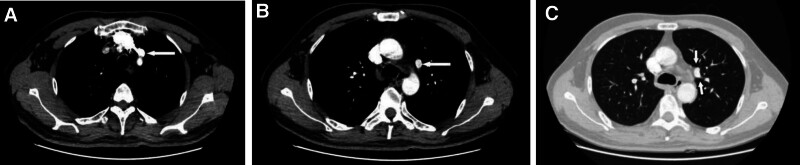
CT confirmed a partial anomalous pulmonary vein connection of the left upper lobe. (A, B) In the mediastinum windows, ectopic drainage of the upper pulmonary veins can be observed in the general direction: blood from the S1 + 2 + 3 vein in the left upper lobe of the lung returns to the left cephalobrachial vein along the superior border of the aortic arch. (C) Variant pulmonary vein bifurcation is seen in lung window. CT = computed tomography.

**Figure 3.**
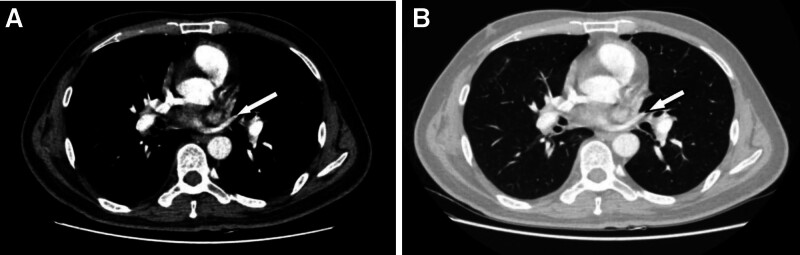
CT showed a special variant of the S4 vein. (A, B) Lung and mediastinum window shows a separate regurgitation of the S4 segment of the left upper lobe of the lung into the left atrium (indicated by the white arrow). CT = computed tomography.

**Figure 4.**
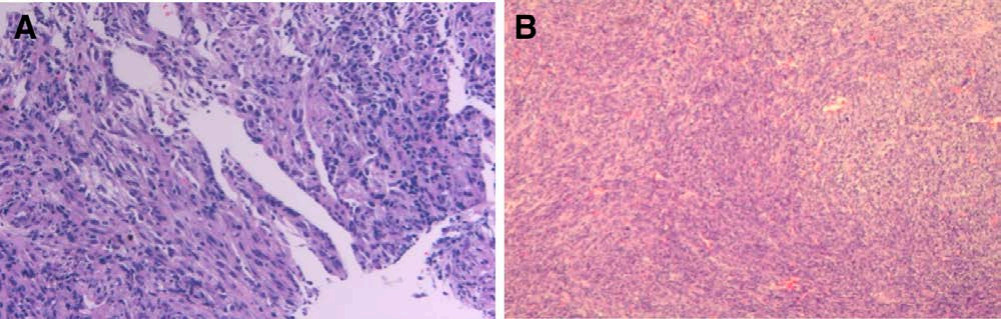
Preoperative and postoperative hematoxylin and eosin staining of pulmonary sarcomatoid carcinoma. (A, B) Spindle cell malignancy with interstitial hemorrhage and necrosis, in which nuclear division images are occasionally visible, is consistent with a sarcomatoid carcinoma.

A 55-year-old man, was found an abnormal shadow in the left lower lobe during a regular checkup. Computed tomography (CT) revealed a 4.1 × 3.6 cm mass in the left lower lobe (Fig. 1A, B). Incidentally, an anomalous pulmonary vein draining from the left upper lobe into the left brachiocephalic vein was also identified (Fig. 2A–C). Additionally, the lingula segment (S4) vein of the left upper lobe was shown to drain into the left atrium (Fig. 3A, B). The patient had no history of cardiac surgery. Preoperative transthoracic echocardiography demonstrated normal biventricular size and function, with an estimated left ventricular ejection fraction of 67%. Right ventricular size was within normal limits, and pulmonary artery systolic pressure was estimated to be 24 mm Hg. No signs of right heart volume overload or pulmonary hypertension were observed. As the mass was suspected to be lung cancer, we considered it likely to be resectable lesion. Preoperative CT-guided fine needle aspiration biopsy indicated the possibility of sarcomatoid carcinoma (Fig. 4A). A curative video-assisted thoracoscopic left lower lobectomy with systemic lymph node dissection was subsequently performed. Arterial gas analysis revealed a preoperative arterial partial pressure of oxygen (PaO_2_) of 69.0 mm Hg and partial pressure of carbon dioxide of 30.5 mm Hg. Postoperatively, the PaO_2_ improved to 75.0 mm Hg and the partial pressure of arterial carbon dioxide decreased to 26.2 mm Hg. Spirometry showed normal pulmonary function. The patient had a satisfactory postoperative course with no signs of heart failure. Histopathological analysis confirmed the diagnosis of sarcomatoid carcinoma, with a tumor size of 4.3 × 3.5 cm (Fig. 4B). From 14 months of follow-up, the patient remained in good health with no evidence of recurrence.

## 3. Discussion

Surgical correction of PAPVC is generally recommended in patients with a ratio of pulmonary and systemic circulation flow >2.0, regardless of the presence of associated cardiac defect.^[4]^ If left untreated, significant left-to-right shunting may lead to right ventricular dilatation, pulmonary hypertension, prominent right pulmonary vasculature, and central pulmonary artery dilatation, particularly in longstanding left-sided PAPVC.^[5]^ Although the right side is the most commonly involved in PAPVC, as shown in autopsy, surgical, and angiographic studies,^[3]^ more recent imaging-based evaluation and reviews of pneumonectomy cases have reported comparable frequencies on both sides.^[6]^ Clinical manifestations such as fatigue, dyspnea, syncope, atrial arrhythmias, right heart failure, and pulmonary hypertension are uncommon, owing to the typically small left-to-right shunts volumes in most cases.^[7]^ Some studies suggest that when PAPVC is present in different lobe from the one being resected, a massive lung resection may increase shunt volume and precipitate right heart failure.^[3]^ Several case reports have described surgical correction of PAPVC prior to lung resection. For instance, Sakurai et al,^[8]^ reported the use of cardiopulmonary bypass to repair a right-sided PAPVC, while Takei et al,^[9]^ described a case in which an anomalous pulmonary vein from the left upper lobe, draining into the left brachiocephalic vein, was separated and anastomosed to the stump of the inferior pulmonary vein.

While these cases required correction of contralateral PAPVC, our case illustrates that ipsilateral and asymptomatic variants may not necessitate intervention. In our study, a video-assisted thoracoscopic lobectomy of the left lower lobe was successfully performed in a patient with ipsilateral PAPVC originating from the left upper lobe. The anomalous left superior pulmonary vein drained directly into the left brachiocephalic vein, a variant not previously reported in the literature to our knowledge. Our decision not to surgically correct the PAPVC in this case due to several reasons: the anomalous pulmonary vein was ipsilateral to the resected lobe, which minimizes the risk of increasing left-to-right shunting after lobectomy; the patient has no clinical or echocardiographic signs of right heart strain or pulmonary hypertension; the ratio of pulmonary and systemic circulation flow is not significantly elevated; and partial preservation of normal pulmonary venous drainage, with the lingular vein draining into the left atrium, further reduced the hemodynamic impact of the PAPVC. The decision not to correct the PAPVC was further supported by the patient’s postoperative physiological response. Arterial blood gas analysis showed an improvement in oxygenation (PaO_2_ increased from 69 to 75 mm Hg) and a reduction in carbon dioxide retention (partial pressure of arterial carbon dioxide decreased from 42 to 38 mm Hg), indicating enhanced gas exchange and ventilatory efficiency following lobectomy. These changes suggest that the residual left-to-right shunt was either insignificant or functionally well compensated, and that the removal of the affected lobe did not lead to any measurable deterioration in pulmonary function. Although the anomalous connection in this case was not associated with any apparent hemodynamic burden or symptoms, its preoperative identification had practical and educational value. Had the anomalous vein not been recognized, there might have been a risk of misinterpretation or inadvertent ligation during surgery, especially in more complex or contralateral cases. Thus, even in asymptomatic patients, recognizing such rare PAPVC patterns helps optimize surgical planning and prevent intraoperative complications. Reporting this case adds to the collective anatomical knowledge and reinforces the importance of vigilance for PAPVC, particularly in thoracic oncologic procedures.

## 4. Conclusion

This case highlights the safe and successful management of pulmonary sarcomatoid carcinoma coexisting with ipsilateral PAPVC without the need for surgical correction. Careful review of preoperative contrast-enhanced CT imaging is essential when evaluating patients with lung cancer, especially when abnormal pulmonary venous drainage is suspected. Our experience suggests that in asymptomatic patients with ipsilateral and hemodynamically insignificant PAPVC, lobectomy alone may be a sufficient and appropriate treatment approach.

## Author contributions

**Conceptualization:** Xiayimaierdan Yibulayin, Xiaohong Sun.

**Writing – original draft:** Xiayimaierdan Yibulayin.

**Writing – review & editing:** Guliniaer Feida.
